# Submucosal tunneling endoscopic resection of a rare gastric hemangioma

**DOI:** 10.1055/a-2523-2575

**Published:** 2025-02-06

**Authors:** Ka-Kin Sze, Wai-Ming Chau, Yiu-Keung Ma, Michael Kin-Kong Li

**Affiliations:** 1 36658 Medicine and Geriatrics, Division of Gastroenterology and Hepatology, Tuen Mun Hospital, Hong Kong, Hong Kong

Gastric hemangioma is a rare gastric tumor, with its incidence reported to be 1.6% 1 . Gastrointestinal bleeding is one of its presenting symptoms 2 . Treatment was traditionally by surgical resection 3 . Recently, there have also been case reports of gastric hemangiomas being resected by endoscopic methods including endoscopic submucosal dissection (ESD) 4 5 . Herein, we present a case of a gastric cavernous hemangioma being resected using submucosal tunneling endoscopic resection (STER).

A 73-year-old man underwent an upper gastrointestinal endoscopy for anemia (hemoglobin level 9 g/dL), which showed a 2-cm subepithelial lesion with bluish discoloration at the anterior antrum ( Fig. 1 ). Endoscopic ultrasound (EUS) was subsequently performed and revealed that the lesion was at layer 3 and was showing heterogeneous echogenicity and suspected cystic spaces ( Fig. 2 ). It was decided to proceed with endoscopic removal of the lesion using STER.

**Fig. 1:**
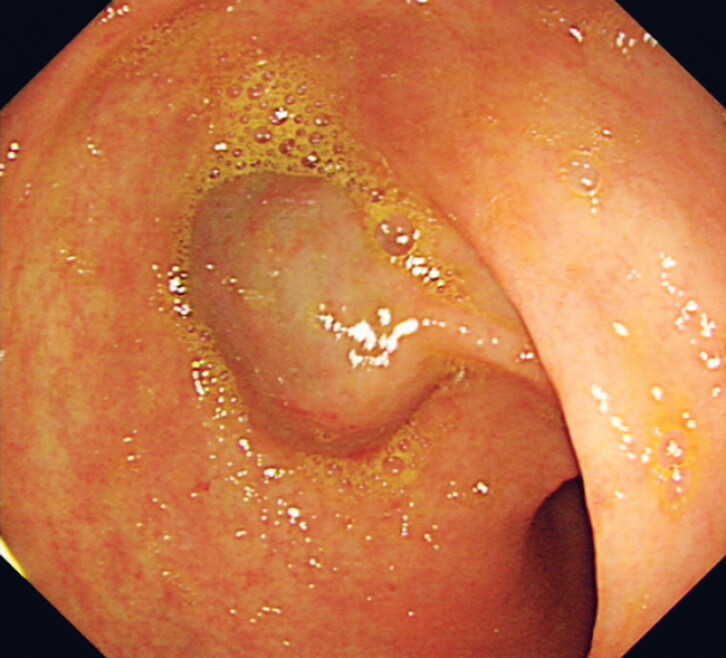
Endoscopic view of the 2-cm lesion showing a bluish discoloration.

**Fig. 2:**
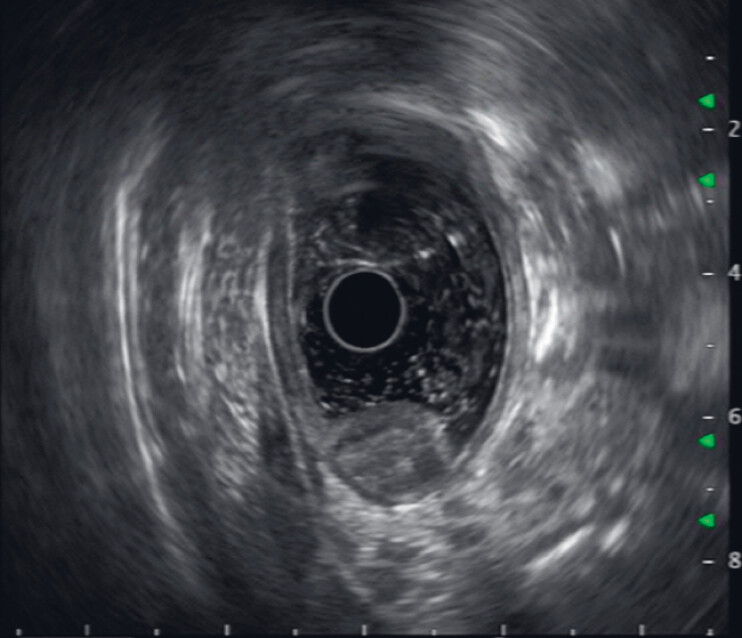
Endoscopic ultrasound view of the lesion showing heterogeneous echogenicity.

The entry of the tunnel was created around 3 cm proximal to the lesion. During the dissection along the tunnel, more vessels were encountered when getting closer to the lesion, with precoagulation of large vessels performed to help maintain a visible endoscopic field. A raspberry-like lesion was noted at the center of the lesion ( Video 1 ). At this stage the procedure needed to be performed with caution as rupture of the cavernous hemangioma could have resulted in rapid blood flow and hemostasis in this situation can be difficult as the hemangioma can be further damaged when receiving thermal energy. The STER procedure was performed uneventfully. Histology revealed a hemangioma. The patient was discharged 2 days after the procedure. A follow-up endoscopy subsequently showed mucosal contraction on the healed wound ( Fig. 3 ) and it was noted that the patient’s hemoglobin level had normalized to 13 g/dL.

Endoscopic resection of a gastric hemangioma is performed using the submucosal tunneling endoscopic resection (STER) technique.Video 1

**Fig. 3:**
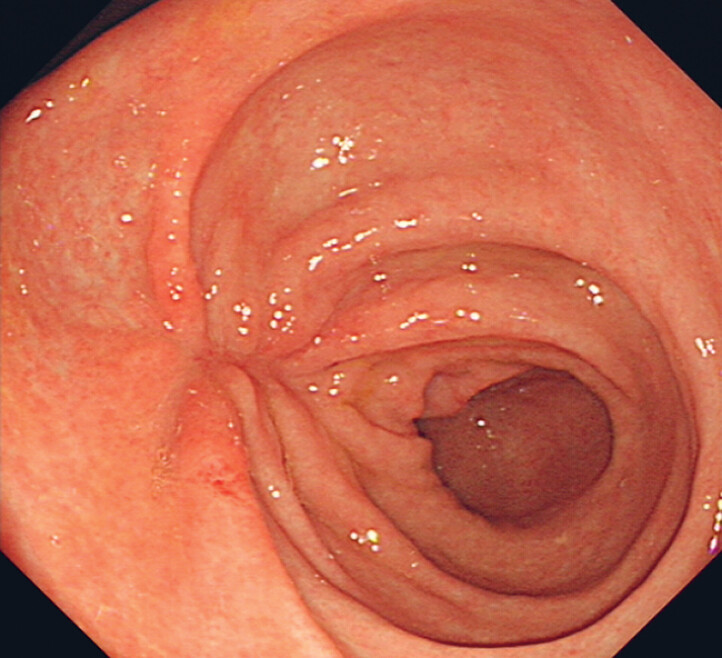
Image from the follow-up endoscopy showing mucosal contraction at the site of the healed wound.

This case demonstrates that STER is feasible for gastric hemangiomas.

Endoscopy_UCTN_Code_TTT_1AQ_2AD_3AZ

## Notes

Endoscopy E-Videos https://eref.thieme.de/e-videos
*E-Videos* is an open access online section of the journal *Endoscopy* , reporting on interesting cases and new techniques in gastroenterological endoscopy. All papers include a high-quality video and are published with a Creative Commons CC-BY license. Endoscopy E-Videos qualify for HINARI discounts and waivers and eligibility is automatically checked during the submission process. We grant 100% waivers to articles whose corresponding authors are based in Group A countries and 50% waivers to those who are based in Group B countries as classified by Research4Life (see: https://www.research4life.org/access/eligibility/ ). This section has its own submission website at https://mc.manuscriptcentral.com/e-videos .
